# Leishmaniasis vectors in the environment of treated leishmaniasis cases in Spain

**DOI:** 10.1111/tbed.14677

**Published:** 2022-08-22

**Authors:** Victoriano Díaz‐Sáez, María Jesús Morillas‐Mancilla, Victoriano Corpas‐López, Javier Rodríguez‐Granger, Antonio Sampedro, Francisco Morillas‐Márquez, Joaquina Martín‐Sánchez

**Affiliations:** ^1^ Department of Parasitology, Faculty of Pharmacy University of Granada Granada Spain; ^2^ Department of Microbiology and Parasitology Hospital Universitario Virgen de las Nieves Granada Spain

**Keywords:** human leishmaniasis, indoors/outdoors, *Leishmania infantum*, patient households, qPCR, sand fly diversity, Spain, vector density

## Abstract

Transmission of leishmaniasis in endemic areas is characterized by microfocality related to the presence of the vector. Most entomological studies in southwestern Europe have focused on sylvatic areas and town outskirts, very few have sampled town or urban centres, and no survey has investigated inside households. The aim of this study was to determine the sand fly species diversity and vector density in the surroundings of human leishmaniasis cases compared with environments in which there was no association. Sand flies were captured in 26 households associated with recently treated leishmaniasis patients, 15 neighbouring houses without associated cases, and in others environments. Overall 7495 sand flies belonging to six species were captured. The highest sand fly density was found in farmhouses where there is a great availability of blood sources and breeding sites. In the environment of human leishmaniasis cases, *Sergentomyia minuta* was the most prevalent species followed by *Phlebotomus perniciosus*. Nevertheless, lower *Leishmania infantum* infection rates and lower intensity of infection were detected in *S. minuta* sand flies than in *P. perniciosus*. The density of *P. perniciosus* in households with recently treated leishmaniasis patients varies between 0 and 108 sand flies per light trap/night, with the maximum values corresponding to farmhouses. This species appears to be adapted to both indoors and outdoors domestic biotopes, including urban households.

## 1 INTRODUCTION

Leishmaniasis is a disease caused by protozoan parasites of the genus *Leishmania* that is transmitted through the bite of infected female sand flies. The presence of sand fly vectors is considered the main risk for the occurrence of leishmaniasis in an area. Non‐vectorial transmission routes such as blood transfusions, needle reuse or sexual and vertical transmission have barely been reported in endemic areas, but some studies show evidence of increased risk posed by these alternative routes (Jiménez‐Marco et al., 2016; Martín‐Sánchez, Torres‐Medina, et al., 2020; Morillas‐Márquez et al., 2002; Pineda et al., 2001, 2002). Vertical transmission appears to play an important role in the spread of leishmaniasis in prolific animal hosts (Martín‐Sánchez, Torres‐Medina, et al., 2020). Leishmaniasis is caused by *Leishmania infantum* in southwestern Europe, where human incidence is low despite the high prevalence found in dogs, its main domestic reservoir. Studies on risk factors for human infection with *L. infantum* have yielded conflicting results, but there is the generalized concept that owners of infected dogs and household members could be at high risk of infection, at least in the Americas (Belo et al., 2013).

*Leishmania infantum* infection in humans leads to clinical disease in only a fraction of all those infected (Aliaga et al., 2019; Pérez‐Cutillas et al., 2015; Riera et al., 2008), but the role of infected people without clinical signs in the epidemiological cycle is not fully elucidated (Molina et al., 2020).

An increasing number of reports suggest that leishmaniasis affects many mammalian species in Europe, both domestic animals and wildlife, as recently reviewed by Cardoso et al. (2021). Wildlife is a major source of infection and interactions with domestic cycles can occur; therefore, understanding the role that wildlife plays in the epidemiology of leishmaniasis will help design measures to reduce prevalence as potential mitigation strategies.

Human intervention in many areas leads to a change in the *Leishmania* transmission cycle by increasing exposure to infected vectors and susceptible reservoirs, or facilitating the interaction between wild and domestic transmission cycles. Different factors related to the urbanization and changes in land use have been involved in the re‐emergence of leishmaniasis in Madrid area, favouring the parasite transmission among Iberian hares and to a lesser extent wild rabbits of the green areas surrounding the municipalities affected by the outbreak (Molina et al., 2012).

A One Health approach tackling zoonotic sand fly‐borne diseases through new approaches in public, veterinary and environmental health may offer several advantages and new options to help control these diseases. Effective control of *L. infantum* transmission requires integrated approaches focusing on all the indirect sources involved in the transmission, the parasite and, importantly, the sand fly vector (Dantas‐Torres et al., 2019).

Three sand fly species of *Larroussius* subgenus have been implicated in the transmission of *L. infantum* in the Iberian Peninsula and southern France: *Phlebotomus perniciosus*—the main vector—, *Phlebotomus ariasi* and *Phlebotomus langeroni* (Alcover et al., 2014; Barón et al., 2011; Branco et al., 2013; Cotteaux‐Lautard et al., 2016; Gálvez et al., 2010; González et al., 2021; Izri et al., 1992; Maia et al., 2013; Martín‐Sánchez et al., 1994; Morillas Márquez et al., 1983; Muñoz et al., 2021; Sáez et al., 2018). *Phlebotomus longicuspis* presence in southern Spain has been ruled out (Martín‐Sánchez et al., 2000; Pesson et al., 2004). Changes in sand fly distribution are essential to determine the potential expansion of leishmaniasis risk areas (Ballart et al., 2012; Díaz‐Sáez et al., 2021; Maroli et al., 2008). In addition, changes in sand fly distribution at the local level could condition the patchy distribution of human leishmaniasis cases in endemic areas. Updated data on vector species at peridomestic level, both intra and peridomiciliary, are necessary for a good understanding of the *L. infantum* transmission dynamics. Therefore, our objective was to determine the sand fly species diversity and vector density in the surroundings of human leishmaniasis cases compared with others environments in which there was no association with human leishmaniasis cases.

## 2 MATERIALS AND METHODS

### 2.1 Study area

The present study was carried out in Granada province (Spain), in the southeast of the Iberian Peninsula (Geographical coordinates of the polygon centroid: 36°15′N 3°15′W) at an average altitude of 1070 m above sea level (0‐3478 m a.s.l.). It has a continental Mediterranean climate in most of the territory, but there is also a mountain climate in the highest area, and subtropical on the coast. The population estimate in 2020 was 919,168 with an average density of 72.8 inhabitants/m^2^. Approximately 25% of the population lives in the city of Granada, the most populated city and the capital of the province. The study area comprises 11 natural geographic regions that can be further subdivided into 174 municipalities, 87% of which have population smaller than 10,000 inhabitants. The economic activity is mainly based on agriculture.

Leishmaniasis has been considered endemic in certain areas of Granada province since 1913 when the first visceral leishmaniasis (VL) case was identified in a child. Human and canine leishmaniasis (CanL), cryptic or symptomatic, are present in the whole province. The annual global incidence of autochthonous human leishmaniasis in Granada province showed an increasing linear trend from a baseline of 0.12 (one case) in 2003 to a peak value of 3.93 (36 cases) in 2016 (Martín‐Sánchez, Rodríguez‐Granger, et al., 2020). CanL prevalence is not uniform across the province, but it is globally high (49.9% prevalence estimated by PCR and 23.7% seroprevalence). High prevalence of *L. infantum* infection has also been found in other animal species in the province of Granada, such as wild rabbits (21%), cats (26%) and wild rodents (27%) by direct techniques, mainly PCR (Díaz‐Sáez et al., 2014; Martín‐Sánchez et al., 2007, 2009; Martín‐Sánchez et al., 2021; Morales‐Yuste et al., 2012; Navea‐Pérez et al., 2015).

### 2.2 Study design

Leishmaniasis is a notifiable disease in Andalusia. Two university hospitals admit all patients presenting with clinical leishmaniasis in Granada province. A prospective study was performed through active surveillance between 2014 and 2016 by the Parasitology, Dermatology, Internal Medicine and Pathological Anatomy services of both hospitals. We assessed clinical presentation and patient characteristics such as age, sex, HIV status and other immunological disorders. The diagnostic methods used were microscopy, in vitro culture, PCR, urine antigen detection and serology (Martín‐Sánchez, Rodríguez‐Granger, et al., 2020). The study and its procedures were explained to the patient and/or their relatives. Written consent was obtained from the enrolled families to capture sand flies at the patient's home.

### 2.3 Sand fly collection and morphological identification

Sand flies were captured using CDC light traps and sticky traps (21 × 29.5 cm paper sheets covered in castor oil) in the environment of (1) 24 recent human leishmaniasis cases occurred between 2015 and 2016, (2) six cryptic leishmaniasis cases found among blood donors, (3) six past cases of human leishmaniasis occurred before 2015 and (4) seven farmhouses without associated human cases. Captures were performed during September and October in 2015 and 2016. Traps were left for one night without rain or wind.

A total of 118 CDC traps (58 placed indoors and 60 outdoors) were placed in dwellings and farms for intradomiciliary survey. When possible, indoor rooms and outdoor spaces (patios and terraces) were sampled. Sand fly genitalia and head were dissected and mounted for species identification.

Nine Hundred Forty‐five sticky traps (total surface 117 m^2^) were placed in dwellings/farms walls and holes, and spread across the town or district. Individuals were taken with care from the surface of sticky papers using a brush soaked in 96° alcohol and subsequently stored in 70% alcohol. Later, the entire specimens were placed in Marc André solution and heated to boiling point, and finally mounted on slides under a coverslip using Berlese solution.

Morphological identification was performed using taxonomic keys and were mainly based on the observation of male genitalia and female spermathecae.

### 2.4 Determination of parasite loads by qPCR

MasterPure DNA Purification Kit (Epicentre, Madison, WI, USA) was used for DNA extraction from female sand flies. DNA purification protocols provided by the manufacturer were used. Briefly, each sample was disrupted using a small pistil, protein was removed using proteinase K and the DNA was concentrated using isopropanol precipitation. DNA quality and quantity were determined spectrophotometrically (Nanodrop 2000c, Thermo Scientific). Extraction controls were used to ensure that the DNA was not cross‐contaminated: the extraction process was simultaneously applied to test‐tubes containing sterile water as well as to the biological samples. The extracted DNA was kept at −20°C. Separately, DNA was also extracted from cultured *Leishmania* spp. reference strains: *L. infantum* (MCAN/ES/91/DP204, MHOM/ES/08/DP532 and MHOM/ES/14/DP581), *Leishmania tropica* (MHOM/MA/88/LEM1314 and MHOM/MA/88/LEM1452) and *Leishmania major* (MHOM/MA/81/LEM265, MRHO/SU/59/LEM129 and MHOM/IL/81/Friedlin).

GRANALEISH Multiplex qPCR (University of Granada, Spain) (Gijón‐Robles et al., 2018) can differentiate between *L. infantum*, *L. tropica* and *L. major* and allows quantification of the parasite load. Primers and the three Taqman probes were provided by the manufacturer. The following thermal profile has been used: 10 min at 95°C, then 36 cycles of 30 s at 95°C and 60 s at 60°C. The number of parasites in every qPCR reaction was calculated through the interpolation of the cycle threshold (Ct) value in a standard curve.

### 2.5 Data analysis

Density (number of specimens per trap and night when using CDC light traps; number of specimens per square meter of trap for sticky traps) and relative abundance (number of specimens of a given species over the total number of captured sand flies expressed as percentage) were calculated for all species identified. Software package IBM SPSS Statistics version 21.0 was used for the statistical analysis.

## 3 RESULTS

We studied the household and family environment of 24 leishmaniasis cases diagnosed between 2015 and 2016 (13 VL, eight cutaneous leishmaniasis, two mucosal leishmaniasis and one cryptic leishmaniasis case with kidney injury). In addition, towns where human leishmaniasis and cryptic leishmaniasis cases had been diagnosed before 2015 were also investigated. As a reference, the captures made in farmhouses without associated human cases were used. Overall, 7495 sand flies were captured of which 4721 were male specimens (63.0%) and 2774 females (37.0%). A larger number of sand flies were captured outside using sticky traps (4521; 60.3%) and the rest using CDC light traps (2974; 39.7%) out of which 1459 (595 females) were captured inside and 1515 (635 females) outside households and farmhouses (patios and terraces). Six species were identified among captured sand flies: *Sergentomyia minuta* was the most abundant species (4510; 60.17%), followed by *P. perniciosus* (2299; 30.67%), *Phlebotomus sergenti* (358; 4.78%), *Phlebotomus papatasi* (284, 3.79%), *P. ariasi* (42; 0.56%) and *P. langeroni* (2; 0.03%).

Most sand flies (57.5%) were captured in the environment of 2015–2016 leishmaniasis cases (4309; 1573 females) where a greater collection effort was made, followed by animal farms without associated cases (2283; 834 females), the environment of cryptic leishmaniasis cases among blood donors (631; 221 females) and the environment of leishmaniasis cases occurred before 2015 (272; 146 females) (Table 1). Table 1 also shows the density and abundance of *P. perniciosus*, *P. ariasi*, *S. minuta* and all species, in the four environments considered. Linear regression showed statistically significant differences between the density of *P. perniciosus* and all sand fly species in the farms with respect to the rest of the environments (*p* < .05). No statistically significant differences were detected between towns/districts within each environment type.

**TABLE 1:** Number (N), density (D)—Sand flies/m^2^/night and sand flies per LT trap/night—and abundance (A) of *Phlebotomus perniciosus*, *Phlebotomus ariasi*, *Sergentomyia minuta* and all species including *Phlebotomus sergenti*, *Phlebotomus papatasi* and *Phlebotomus langeroni*, in the four environments considered

Environment type and number of traps		*Phlebotomus perniciosus* (♀)	*Phlebotomus ariasi* (♀)	*Sergentomyia minuta* (♀)	Total sand flies (♀)
Environment of 24 leishmaniasis cases occurred between 2015–2016 637 AT (*n* = 36*); 89 LT (*n* = 36*)	N D A	940 (325) 4.8/m^2^; 6.3/LT 21.8	32 (20) 0.06/m^2^; 0.3/LT 0.7	3078 (1126) 28.1/m^2^; 9.7/LT 71.4	4309 (1573) 33.6/m^2^; 18.6/LT
Environment of six cases of human leishmaniasis occurred before 2015 100 AT (*n* = 6*); 10 LT (*n* = 4*)	N D A	70 (50) 3.1/m^2^; 3.6/LT 25.8	2 (1) 0 /m^2^; 0.2/LT 0.7	147 (76) 9.1/m^2^; 3.9/LT 54.0	272 (146) 14.2/m^2^; 10.7/LT
Environment of six cryptic leishmaniasis cases 110 AT (*n* = 7*); one LT (*n* = 1*)	N D A	204 (31) 14.8/m^2^; 2/LT 32.3	3 (2) 0.22/m^2^; 0/LT 0.5	388 (179) 28.5/m^2^; 0/LT 61.5	631 (221) 46.2/m^2^; 2/LT
Animal farms (7) without associated human cases 98 AT (*n* = 7*); 18 LT (*n* = 7*)	N D A	1085 (310) 33.2/m^2^; 37.9/LT 47.5	5 (0) 0.08/m^2^; 0.2/LT 0.2	897 (353) 47.0/m^2^; 18.2/LT 39.3	2283 (834) 87.4/m^2^; 67.8/LT
Total sand flies 945 AT, 118 LT	N D A	2299 (716) 8.7/m^2^;10.8/LT 30.7	42 (23) 0.08/m^2^; 0.3/LT 0.6	4510 (1734) 28.1/m^2^; 10.4/LT 60.2	7495 (2774) 38.6/m^2^; 25.2/LT

*Note*: *n*, number of stations sampled.

Abbreviations: AT, adhesive trap; LT, CDC light trap.

* Some stations were sampled with both types of traps while others were only sampled with one of them; thus, some AT and LT stations are the same station, while others are different.

Sand flies were captured in 41 households, 26 associated with 24 leishmaniasis cases diagnosed between 2015 and 2016 (two cases had two family homes and both were inspected), and 15 neighbouring houses without associated cases, which do not include the seven farms used as reference. The densities of sand flies of subgenus *Larroussius* species, mainly *P. perniciosus*, captured inside and outside these households are shown in Table 2. *Larroussius* sand flies densities were not significantly different between household types using either logistic regression (*p* ≥ .400) or ANOVA comparison of the means (*p* ≥ .280). Fisher's test did not detect significant differences in the medians either (*p* ≥ .411).

**TABLE 2:** Densities (sand flies/m^2^/night and sand flies per LT trap/night) of subgenus *Larroussius* sand flies domestic captures in the environment of 24 leishmaniasis cases occurred between 2015 and 2016. Two cases had two family homes and both were inspected; the 15 households without human case are neighbouring houses which do not include the seven animal farms used as reference. Almost all the individuals belonged to *Phlebotomus perniciosus* species. The presence of *Phlebotomus ariasi* was very low, and only one individual of *Phlebotomus langeroni* was captured

Households	Trap type and location	Minimum	Maximum	Mean	Standard deviation	Median
With human case N = 26	LT indoors	0 (7)	107.50	14.41	33.28	0.5
	LT outdoors	0 (7)	24.66	3.89	7.57	0.25
	LT total	0 (7)	107.50	10.19	25.09	0.5
	AT	0 (6)	25.80	4.12	6.54	0.65
Without human case N = 15	LT indoors	0 (6)	7.7	1.10	2.54	0
	LT outdoors	0 (2)	6.0	1.59	2.26	0.67
	LT total	0 (4)	7.7	2.13	2.94	0.46
	AT	0 (2)	8.0	1.70	3.11	0.66
All households N = 41	LT indoors	0 (13)	107.50	9.80	27.44	0
	LT outdoors	0 (9)	24.66	3.03	6.16	0.67
	LT total	0 (11)	107.50	7.67	21.07	0.46
	AT	0 (8)	25.80	3.56	5.96	0.66

*Note*: N, number of sampled households. Values in parenthesis in minimum column represent number of households.

Abbreviations: AT, adhesive trap; LT, CDC light trap.

A positive correlation was detected between *Larroussius* sand fly densities captured with CDC traps indoors and captured outdoors (Pearson correlation = 0.703, *p* = .016), as well as between the *Larroussius* sand fly densities trapped outdoors with CDC and those captured with adhesive traps (Pearson correlation = 0.775, *p* = .001).

In the city of Granada, eight dwellings were investigated: seven associated with leishmaniasis cases and one no‐case. The sand fly densities of subgenus *Larroussius* species, mainly *P. perniciosus*, captured inside and outside these households according to its location in Granada city or in the towns of the province are presented in Table 3. Using logistic regression, no differences were detected between the densities of sand flies of *Larroussius* subgenus captured in dwellings located in towns and those located in Granada city (*p* > .299). With Fisher's test, significant differences were detected between median values from Granada city and towns corresponding to outside CDC captures (*p* = .037), whereas no differences were detected in the other (*p* ≥ .32).

**TABLE 3:** Densities of subgenus *Larroussius* sand flies urban and rural captures in the environment of 24 leishmaniasis cases occurred between 2015 and 2016. Two cases had two family homes and both were inspected; the 15 households without human case are neighbouring houses which do not include the seven animal farms used as reference

Households	Trap type and location	Minimum	Maximum	Mean	Standard deviation	Median
Granada city N = 8	LT indoors	0 (3)	8	1.88	2.92	0
	LT outdoors	0 (4)	0.25	0.05	0.11	0.61
	LT total	0 (3)	8	1.45	2.73	0.24
	AT	0 (4)	4.71	0.94	2.11	0
Towns N = 33	LT indoors	0 (10)	107.5	12.72	31.77	0.5
	LT outdoors	0 (5)	24.66	3.82	6.73	1
	LT total	0 (8)	107.5	9.74	24.03	0.59
	AT	0 (4)	25.8	4.18	6.44	0.7
All N = 41	LT indoors	0 (13)	107.50	9.80	27.44	0
	LT outdoors	0 (9)	24.66	3.03	6.16	0.67
	LT total	0 (11)	107.50	7.67	21.07	0.46
	AT	0 (8)	25.80	3.56	5.96	0.66

*Note*: N, number of sampled households. Values in parenthesis in minimum column represent number of households.

Abbreviations: AT, adhesive trap; LT, CDC light trap.

*Leishmania infantum* DNA was quantified through qPCR in 132 field‐captured female sand flies: 98 *S. minuta* and 35 *P. perniciosus*. Lower infection rates and parasite loads were detected in *S. minuta* sand flies (28.6%: two showed low loads [10–100 parasites/sand fly] and 26 showed very low parasite loads [<10 parasite/ sand fly]) than in *P. perniciosus* (34.3%: 3 showed very high loads [>10.000 parasites/sand fly], four showed low loads [10–100 parasites/sand fly] and five showed very low parasite loads [<10 parasite/sand fly]).

## 4 DISCUSSION

The transmission of leishmaniasis is characterised by focality, usually linked to certain ecosystems; the drivers of this focality are not always understood but are related to the presence of the vector. Furthermore, within these ecosystems, sand fly populations are the highest in particular microhabitats that can consequently cause a microfocal disease distribution. Many reports have investigated the links between environmental factors and changes in *L. infantum* vector distributions in southwestern Europe, which could result from a combination of human activities and natural phenomena such as climate warming (Alcover et al., 2014; Ballart et al., 2012; Barón et al., 2011; Gálvez et al., 2010; Prudhomme et al., 2015; Risueño et al., 2017). Most of these entomological surveys focus primarily on sampling between towns or at town limits, mainly on embankments although also farms and cottage houses. Few studies have performed surveys in towns and none inside patient households. Of the three potential vectors of *L. infantum* in southwestern Europe, *P. perniciosus* is the main cause of transmission due to its wider distribution and density. The presence of *P. perniciosus* and its density on embankments is greater between towns than in other locations such as town boundaries or town centres (Barón et al., 2011; Gálvez et al., 2010). However, when the characteristics of the sampling sites are not as homogeneous as the embankments and walls, the highest density is reached in biotopes that include domestic animal shelters, which tend to be more frequent in peridomestic environments located at town limits (Branco et al., 2013; Bravo‐Barriga et al., 2016; Maia et al., 2013). *Phlebotomus perniciosus* density has been also assessed in periurban residential estates showing widely variable density data that are greater in adjacent non‐urbanized sites (Muñoz et al., 2021) and in peri‐urban green areas, as in the focus of leishmaniasis in Madrid where it was the only vector and showed high densities and infection rates (González et al., 2017). To the best of our knowledge, this is the only study in which sand flies captures are made in European households associated with leishmaniasis cases due to *L. infantum*. Other similar surveys have been carried out in India and Nepal, where *Leishmania donovani* is endemic (Picado et al., 2010); and in *L. tropica* endemic regions in Morocco (Gijón‐Robles et al., 2018).

Five sand fly species commonly found in southwestern Europe (*P. perniciosus*, *P. papatasi*, *P. ariasi*, *P. sergenti* and *S. minuta*) and the frequently overlooked, *P. langeroni* were found in the biotopes studied in the present study (Barón et al, 2011; Morillas Márquez et al., 1983; Sáez et al., 2018)*. Phlebotomus perniciosus* was the most abundant and densest *Larroussius* species in households with recently treated leishmaniasis patients as in the other three environments, while *P. ariasi* abundance and density were generally low, in agreement with the preference of this species for humid or sub‐humid areas (Ballart et al., 2012) as opposed to the semi‐arid or arid character of the sampled areas. Despite this fact, *P. ariasi* covers a wide geographic range in the western Mediterranean showing a high intra‐specific diversity (Franco et al., 2010). *Phlebotomus langeroni* is associated with the existence of rabbit burrows in immediate surrounding areas of the capture sites, a necessary condition for the presence of this species (Sáez et al., 2018). The very low density of this sand fly species in domestic environments seems to indicate that although *P. langeroni* is a competent vector, its involvement in transmission in this environment is negligible and would be limited to the sylvatic transmission cycle.

The highest sand fly density was found in farmhouses where there is a great availability of vertebrates as blood sources for females to mature the eggs, abundant organic matter to find breeding sites and resting sites for adult sand flies to shelter during the day. *Phlebotomus perniciosus* is an opportunistic biter of a wide range of hosts, including humans (Bravo‐Barriga et al., 2016; González et al., 2017), and farmhouses show optimal suitability for this vector, reaching in these microenvironments the highest values of relative abundance.

*Sergentomyia minuta* was the most prevalent species in the study overall and in the environment of leishmaniasis cases, similar to other surveys we have carried out in peridomestic and sylvatic biotopes in the province (Barón et al., 2011). The herpetophilic *S. minuta* prefers holes within rocks or walls because these biotopes allow sympatry with reptiles. Sticky traps were 2 to 4 times more effective for this species than CDC traps in all studied environments because *S. minuta* does not exhibit phototaxis, and trapped insects could only be random catches that reveal their presence in the biotope. Nevertheless, *S. minuta* density in the environment of leishmaniasis cases exceeds that of *P. perniciosus* with both trapped methods. Records of *S. minuta* blood meals on mammals, including humans, and detection of *L. infantum* DNA in *S. minuta* are increasing; therefore, the need to explore a potential route of leishmaniasis transmission involving this sand fly species has been suggested (Gonzalez et al., 2020; Pereira et al., 2017; Pombi et al., 2020). The low parasitic loads detected in *S. minuta* compared to *P. perniciosus* captured in the same sites could be more important than the infection rate for the hypothetical vectorial capacity of this sand fly species. Although the infection rate is high, none of the infected *S. minuta* females found had a parasite load greater than 100 parasites/sand fly, while 25% of infected *P. perniciosus* females had very high infection rates with more than 10,000 parasites/sand fly. In this study, in addition to walls and holes, adhesive traps were placed in sewers, finding a high proportion of *S. minuta*. Sewers have been suggested as resting sites for sand flies in urban areas (Galán‐Puchades et al., 2022). The infection intensity of sand flies caught in the wild supports parasite growth and transmission potential, and the quantification of parasite burden in sand flies by qPCR could be suitable for the incrimination of suspected vectors of leishmaniasis. The low parasite loads we found in all *S. minuta* studied contrast with the high parasite loads observed in some *P. perniciosus* females and suggest that *S. minuta* lack high transmission capacity of *L. infantum*.

The densities of *Larroussius* species captured outdoors with CDC traps were correlated with those captured the same night with sticky traps showing that both types of traps are almost equally effective for capturing these species. This is important from a methodological point of view: the use of CDC traps is not recommended in unsafe environments outside homes where they can be stolen; on the other hand, home owners often refuse sticky traps indoors because they fear oil stains.

We did not find significant differences between intradomiciliary captures of *P. perniciosus* performed indoors and captures in patios or terraces, which suggests that *P. perniciosus* is well adapted to both indoors and outdoors domestic biotopes; in addition, females with blood in their guts were collected from both type of catches. The extent of endophily/exophily behaviour of this species is an important research question relating to the relative importance of indoor and outdoor transmission that has implications for prophylaxis and control.

The average density of *P. perniciosus*, captured inside and outside, was highest in households with associated leishmaniasis cases than in those neighbouring houses without associated cases, although no statistically significant differences were detected, probably because the total number of households researched was relatively low. In spite of this limitation, these data should prove useful for the design of a large‐scale study addressing this issue by extending collections to a larger number of dwellings. In this study, the density of *L. infantum* vectors in households with recently treated leishmaniasis patients varied between 0 and 108 sand flies per light trap/night, influenced by factors related to housing conditions, with the maximum values corresponding to farmhouses.

Leishmaniasis due to *L. infantum* is classically considered a rural zoonotic disease; however, and according to our results, *P. perniciosus*, the primary vector of *L. infantum*, can also be found in urban areas, both indoors and outdoors. *Phlebotomus perniciosus* also breeds in these urban peridomestic and domestic environments with enough organic matter to feed larvae. Females need blood meals, and like males, use plant as their main sources of energy and water. The existence of gardens and parterres in urban contexts is widespread in cities from southwestern Europe, and pots are common in houses, being able to provide water and energy to adults and serving as a habitat for larvae.

In conclusion, the highest sand fly density was found in farmhouses, both associated and not associated with leishmaniasis cases, where there is a great availability of blood sources and breeding sites. Houses with associated cases have a higher sand fly density than those without associated cases, although without statistical significance due to the sample size. In the environment of human leishmaniasis cases, *S. minuta* is the most prevalent species but lacks the high *L. infantum* transmission capacity of *P. perniciosus*, the second most abundant and densest species. This species appears to be adapted to both indoors and outdoors domestic biotopes, including urban households.

## CONFLICT OF INTEREST

The authors declare no competing interests.

## AUTHOR CONTRIBUTIONS

Joaquina Martín‐Sánches, Victoriano Díaz‐Sáez and Francisco Morillas‐Márquez conceived and design the research. Joaquina Martín‐Sánches, Victoriano Díaz‐Sáez, Javier Rodríguez‐Granger and Antonio Sampedro were involved in acquisition of local data. Victoriano Díaz‐Sáez, María Jesús Morillas‐Mancilla, Joaquina Martín‐Sánches and Francisco Morillas‐Márquez conducted captures and morphological identification. Joaquina Martín‐Sánches, Victoriano Díaz‐Sáez and Victoriano Corpas‐López analyzed data and conducted statistical analyses. Joaquina Martín‐Sánches and Victoriano Díaz‐Sáez wrote the manuscript. Joaquina Martín‐Sánches, Victoriano Corpas‐López and Francisco Morillas‐Márquez revised the manuscript critically. All authors read and approved the manuscript

## ETHICS STATEMENT

The procedure was approved by the Ethics Committee of the University of Granada.

## Data Availability

The data that support the findings of this study are available from the corresponding author upon reasonable request.
